# Patient preferences for notification of normal laboratory test results: A report from the ASIPS Collaborative

**DOI:** 10.1186/1471-2296-6-11

**Published:** 2005-03-08

**Authors:** Donna M Baldwin, Javán Quintela, Christine Duclos, Elizabeth W Staton, Wilson D Pace

**Affiliations:** 1 Kaiser Permanente Medical Group, Aurora, Colorado, USA; 2 Department of Family Medicine, University of Colorado at Denver and Health Sciences Center, Denver, Colorado, USA; 3 John Snow, Inc. Denver, Colorado, USA

## Abstract

**Background:**

Many medical errors occur during the laboratory testing process, including lost test results. Patient inquiry concerning results often represents the final safety net for locating lost results. This qualitative study sought to identify, from a patient perspective, specific preferences and factors that influence the process of communicating normal (negative) laboratory test results to patients.

**Methods:**

We conducted 30-minute guided interviews with 20 adult patients. Patients were recruited from two practice-based research networks in Colorado that were participating in a medical errors study. A semi-structured interview elicited the participant's experience with and preference for laboratory test result notification. Quantitative descriptive statistics were generated for demographic and preference data. Qualitative results were analyzed by a team of experienced qualitative researchers using multiple styles of qualitative analyses, including a template approach and an editing approach.

**Results:**

Ninety percent of participants wanted to be notified of all tests results. Important issues related to notification included privacy, responsive and interactive feedback, convenience, timeliness, and provision of details. Telephone notification was preferred, followed by regular mail. Electronic notification was perceived as uncomfortable because it was not secure. While 65% preferred being notified by a provider, participants acknowledge that this may be impractical; thus, they wanted to be notified by someone knowledgeable enough to answer questions. Participants do not normally discuss their preferences for test result notification with their providers.

**Conclusion:**

Privacy, responsive and interactive feedback, convenience, and timeliness with detailed information may be critical for patient satisfaction and for improving patient safety, and are features that may be incorporated into emerging communication channels.

## Background

Understanding how patients would like to be notified of laboratory test results is important for improving provider-patient communication, patient satisfaction, and patient safety. Data from Applied Strategies for Improving Patient Safety (ASIPS), a primary care practice-based study of medical errors, indicate a high frequency of errors in laboratory testing and patient notification[1]. Other studies in primary care show similar safety concerns with laboratory testing[2,3]. Preliminary ASIPS data show that laboratory errors were commonly discovered by patient-initiated requests for results. A study of patient notification of emergency department test results found that passive notification – the "no news is good news" approach – was ineffective and potentially dangerous[4]. Thus, ensuring that patients receive all lab results – even normal results – may be an important and last safety net for identifying missing or mishandled laboratory results.

Patients want to be notified of all test results, regardless of whether the results are abnormal [4-8] A few studies have explored patient's preferences for being notified of specific test results, reporting that patients prefer to be notified by telephone for breast biopsy results[9] and normal mammograms[6], and wish to receive timely, detailed, written information for normal pap smear results[10]. We found no studies from primary care concerning notification procedures and patient preferences. To inform future interventions in this area, we elicited patients' thoughts, needs, and preferences regarding test result notification.

## Methods

### Sample

This study was conducted within the Colorado Research Network (CaReNet) and High Plains Research Network (HPRN), two primary care practice-based research networks. CaReNet practices are located mostly in urban / suburban cities in Colorado; HPRN practices are in rural northeast Colorado. We intentionally used these settings as the most optimal example of the setting (primary care clinics) in which we would see the need for normal lab result notification. We purposively sampled study participants based on emerging themes from our analysis. Thus, we intentionally recruited participants who were 18 years of age or older, able to speak and understand English or Spanish, and who had at least one laboratory test at a participating practice within the last year. Laboratory tests were defined as blood or urine tests, pap smears, and biopsies. The aim of our strategy was not to maximize generalizability, but rather to understand the context and conditions under which normal lab result notification does or does not occur. We recruited to the point of saturation or replication of data[11]. All final 20 participants spoke English and gave verbal telephone consent.

### Procedures

Patients were recruited via posters and business cards placed in 12 primary care practices participating in the ASIPS study. ASIPS was a multi-institutional, primary care practice-based project to collect, codify, and analyze data on medical errors[1,12]. Interested patients called an automated call center and left their names and telephone numbers. A research assistant called patients to determine eligibility, obtain consent, and schedule a 30–45 minute interview. An interview was completed by telephone (19 cases) or face-to-face (1 case) and was audio taped.

This study was approved by the University of Colorado Health Sciences Center's institutional review board.

### Measures

#### Demographic information

We collected the patient's age, gender, race, ethnicity, highest level of education, if they had a permanent home address, and access to the Internet and e-mail. Patients were asked if they had personally received results of a test by message left on answering machine, through phone conversation, mail, e-mail, automated telephone call-up system, or via web-based system. Patients ranked these notification methods by their preference.

#### Semi-structured interview

The interview began with questions about the patient's most recent experiences with test result notification (who, what, when, etc.). The interview then shifted to preferences of notification and patient-doctor discussion about notification preferences. Inquiry also elicited patient factors that may affect test result notification.

An in-depth interview provides a narrative understanding of how particular individuals arrive at their experience. Our purpose was to construct a meta-narrative of the interviewees' many stories. This on-going interpretive process informed each subsequent interview in an iterative fashion. For example, during early interviews it appeared that different preferences might be expressed by people with different educational levels. We then stratified our subsequent sample by educational level (more than a high school education vs. high school education or less).

### Data analysis

We used mixed methodology to analyze the data. Quantitative descriptive statistics (frequencies, proportions, etc.) were generated for our demographic and preference variables. All coding and analysis was done by a three-member team that included a physician, a doctorate researcher experienced in qualitative methodology, and a professional research assistant. The use of a multidisciplinary team approach helped limit any personal biases, subjectivity, and preconceptions as well as enhanced our reflexivity process[11]. A commitment to reflexivity resulted in ongoing assessment of subjectivity by the team in all steps of the analyses[13]. Our qualitative analysis was initially guided by multiple styles of qualitative analyses including an initial template approach as outlined by Crabtree and Miller[11] using already published literature on lab result notification as guides. We created an initial a priori template of codes (code manual) and then applied it to the text data. This approach was then followed by editing approach, a technique derived from grounded theory, to identify emerging themes. Members of the research team independently coded a number of pages of the same text to test for both the utility and appropriateness of the codes and the intercoder reliability, which measures correspondence between two or more coders' assessments (84%)[14]. Achieving an acceptable level of intercoder reliability is important for providing basic validation of coding scheme. We modified the code manual to correct for discrepancies and deficiencies. Research team members then identified and sorted segments of text, which allowed further abstractions and emerging codes. ATLAS.ti software facilitated the iterative coding and sorting process[15]. We further sorted related text, producing connections and interpretations.

## Results

In the end, we found no differences qualitatively or quantitatively across participants' educational levels. Thus, all results are reported as a single group. Most (75%) participants were female (see Table 1); 90% indicated they expected to be notified of all test results, regardless if normal or abnormal.

**Table 1:** Demographics of participants

**Demographic Characteristics**	**TOTAL**	**%**
Female	15	75%
		
Race		
White	15	75%
Black	4	20%
Asian	1	5%
Age		
≤ 30	4	20%
31–40	0	0%
41–50	8	40%
51–60	5	25%
61–70	2	10%
≥ 71	1	5%
Education		
Some or graduate of high school	10	50%
Some college	5	25%
College graduate	1	5%
Post graduate education	4	20%
Have access to e-mail & Internet	19	95%
Location of frequent access to e-mail & Internet		
Home	9	45%
Library	4	20%
Multiple sites	5	25%

When exploring the possible modes of lab result notification (phone, message left on an answering machine, mail, e-mail, automated telephone call up system, and web-based systems) we found no differences in preferences by age groups, educational levels, or access to e-mail and the Internet (table 1). Many participants who had access to the Internet or e-mail were open to the idea of retrieving results via this medium only if the security was assured; however, most patients felt that web-based systems and e-mail are not secure. Only a minority of patients interviewed were willing to try an automated telephone call, e-mail, or web-based system.

Patients' preferences seemed to reflect their most recently experienced method of notification. Table 2 shows total number of patients who experienced a particular method of notification within the past year and their preferred method of notification. Most patients experienced and preferred phone or mail notification. All interviewees stated that a message left on an answering machine was not appropriate.

**Table 2:** Participants' experience with and preference for notification methods

**Selected Methods**	**Experienced Method**	**% (n = 20)**	**Preferred Method**	**% (n = 20)**
Message left on answering machine	9	45%	0	0%
Telephone	16	80%	12	60%
Mail	11	55%	3	15%
Automated telephone call-up	0	0%	2	10%
E-mail	0	0%	2	10%
Web-based system	0	0%	1	5%

We identified three emerging themes: (1) *Important Characteristics of Notification: *most notable factors influencing patient preferences in notification; (2) *Patient/Provider Discussion: *the lack of communication between patients and providers around notification preferences; and (3) *Communication Frustration: *challenges encountered during attempted communication between the patient and the practice regarding notification of their results. Below we briefly describe these themes with illustrative quotations from the participants.

### Important characteristics of notification

Important factors that defined patients' concerns around notification were always being notified of results, timeliness, details of the results, responsive and interactive feedback, who should provide the notification, convenience, and assured security/confidentiality.

#### Always notify

Not surprising, almost all patients responded that they wanted to be notified of all results. The "no news is good news" approach is unacceptable to patients.

"Obviously there was a reason to have that diagnostic test, so I'd at least like to know whether it was normal or what."

"To me, no news is worrisome."

#### Timeliness

Patients wanted to receive their results in a timely manner – shortly after the physician or provider receives the results.

"Let me know right away. Don't keep me hanging. Do the test on Friday and if I don't know till Tuesday, I want to know Monday morning. As soon as you find out the results, you let me know the results because this is my body."

#### Details of test results

The amount and detail of information is important in providing the context in which the patient can interpret the results.

"I would like to know what normal means in relation to the general population, so I would certainly like some reference ranges...If there weren't any reference ranges, then I would certainly like to know that a test...came back negative and what negative meant."

#### Responsiveness & interactive feedback

The test result was only half of the information participants want. They also wished to discuss what the results mean for them. In some cases, patients are left wondering about the "next steps." A patient may feel confused if someone is not available during the notification process who can discuss what the test means.

"Well, the information on what I can and what I can't do, you know. I mean I don't know what I can do at this point."

"The thing I do like is that we can actually talk about the numbers and I can see where it's at because he just gives me, we just look at the sheet together...I like that."

#### Who should notify

Most patients recognize that providers are too busy to attend to all normal test results. They were comfortable having someone else notify them, but preferred that this person be knowledgeable enough to answer questions. Many patients indicated that notification by receptionists, who they felt were not knowledgeable enough to answer questions, is unacceptable. When asked specifically the preferred role of a notification person the responses were: 65% provider and 15% nurse; 20% were not concerned with who notifies.

"Ok, this test result is normal but I still have this pain, what alternative do I have now. Where do I go from here and a physician would be the best person to be able to explain it to me, rather than a physician assistant or a nurse or an administrator or something like that."

"Well, it doesn't have to be the actual doctor. It could be the RN...Somebody who knows about what is going on and if I have a question I could ask that person."

#### Convenience

Patients identified convenience as important to their satisfaction. Calling the office for results can mean long waits to reach a person who notifies them of their result and then longer waits when the results prompt a patient question that cannot be immediately answered.

"That way (using web-based system) I could do it at anytime and it seems more personal and confidential to me because...you can draw up that information at any time when you're ready."

"That's why the mail is so good because I always get the mail everyday."

#### Security/confidentiality

The most persistent issue we uncovered was a patient's privacy and assured confidentiality of test results and diagnoses. Participants seemed hesitant to experiment with alternative notification methods (i.e., web-based methods) if they perceived a possibility of a breach in this trust.

"Who wants the public to read what their values are?...Even if it's normal, I'd rather have it personalized in a sealed envelope."

### Provider discussion

Most patients assumed that their office used a specific system for notification; however, they were not aware of the details of this system. Often patients were told to call for results if not received within specified time, leaving the patients to close the feedback loop. Other patients were told to rely on "no news is good news," although they were uncomfortable with this policy.

The majority of patients indicated that they had not discussed their notification preferences with their provider.

"No, I haven't. I just assumed that was normal and customary procedure...That they only notify you if something is wrong."

"I didn't know they were just going to send me a letter. In the past, I've had the doctors call me. Well, they automatically call [ed]."

### Communication frustration

Two areas of frustration related to communication were identified: lack of follow-through and confusion within the office.

#### Follow-through

Communicating how the provider will notify the patient is important, but equally important is follow-through. Unmet expectations result in patient frustration.

"I told him that he could call me, or the nurse. Either one would be ok, but...it doesn't happen. Even though I asked them face to face, it still hasn't happened."

#### Office confusion

Patients are also frustrated and worried when they try to complete the feedback loop, but find that the practice is unable to provide them the information they need.

"I have to sometimes call in to find out about my results...I wasn't actually notified of the results...so obviously it was my responsibility. I felt it was my responsibility anyway. But...they [the results] seemed to have [been] misplaced. The parties concerned didn't seem to be aware that I had had a particular test and so they couldn't provide me with information...It wasn't until I went back recently and asked about that result, that I was told it was normal."

## Discussion

Our results reinforce other literature suggesting patients want timely[9] and detailed[10,16] information, and want to be notified of all test results, even if normal[5,7]. These results also support the idea that patients prefer clinicians telephone them with lab results[6]. We, other researchers[17,18], and the patients in our study recognize that this plan is often too costly to be practical.

A number of our findings, however, suggest that more than preferring a particular channel of communication, patients prefer a specific manner of communication, features of which could be incorporated into newer communication channels. For example, our participants overwhelmingly preferred a personal telephone call – but only from someone who was knowledgeable enough to answer their questions. They wanted responsive and interactive feedback, *personalized *to their situations.

Many of the patient identified characteristics of notification could be more easily incorporated into a system that allowed for asynchronous communication (communication that doesn't rely on immediate person to person transfer of information.) Nonetheless, few of our participants were willing to try the computerized asynchronous communication methods we asked about: an automated telephone call, e-mail, or web-based system. This finding is similar to others[7], although Ridgeway and colleagues report that patients used and were generally satisfied with an automated phone call up system[19]. Our patients said they would be willing to try a web-based system if they were convinced of security and confidentiality, suggesting that concerns with web systems lie not with the technology, itself, but with the privacy of the information. Notably, privacy was one reason patients wanted a personal phone call. Thus, we suspect that their stated preferences for telephone calls are related to the perceived high level of privacy and interaction available through a synchronous telephone call, while their distrust of websites and e-mail indicate underlying discomfort with the perceived privacy of the technology. Recognizing these tradeoffs may be useful for those who are designing systems to provide test result notification to patients.

Our patients emphasized the importance of receiving results in a timely fashion. Timeliness is a critical feature of notification systems that can significantly affect patient safety; timely recognition of mishandled or misplaced results will increase the practice's ability to correct or mitigate an error. However, timeliness is not an inherent feature of patients' preferred channel of communication: a telephone call. In our experience, providers' and patients' hectic schedules often mean it can take days for successful telephone contact between providers and patients. Again, our patients' focus on timeliness suggests that they are not wedded to the concept of telephone communication; rather, they prefer the perceived timeliness of communication by telephone. Similarly, our participants indicated that convenience was important to them. Again, waiting for a telephone call is not intuitively convenient, though the dramatic increase in cell phones may help alleviate this problem. Perhaps our study participants could not envision a convenient communication method other than phone calls that provide secure, personalized, interactive communication.

A traditional mail-based system (a low-tech asynchronous communication system) was ranked second highest among preferences for notification. This finding detracts from our assumptions that patients focus on notification methods that are timely and interactive; however, it supports our idea that patients want convenient, private, personalized information. Perhaps the three patients who preferred mail notification were more concerned with privacy and convenience than with timeliness and interactivity. Further study is needed to elucidate these findings.

Finally, we found that patients do not discuss with their provider their preferences for notification. Our patients indicated that it never occurred to them that their health care provider lacked a standard procedure for communicating test results to patients. Our findings may be a manifestation of poor communication between patients and providers, which has been shown to be a related to poor patient outcomes and safety issues[20]. More study is needed to explore this example of poor interpersonal communication.

A potential limitation of this study relates to the recruitment, which was limited to patients who had access to call our research line after seeing an advertisement. However, qualitative inquiry rarely uses random sampling. Rather, samples are selected more purposefully and not by the need to generalize or predict but by a need to create deeper understanding or meaning[11]. Thus, studying the narratives of people who called our research line to talk about their experiences is appropriate and adequate. Additionally, we sampled to the point of redundancy; no new information was coming forth by the end of 20 interviews. A second limitation may be a gender bias. Most of our respondents were female. Male experiences may differ and may not be represented by our results. However, considering that the majority of health care utilizers are women, and women are critical in maintaining the health and health care consumption within families [21-23] their experience becomes crucial for primary care service delivery. Finally, the most significant limitation of this study relates to the participants' lack of experience using a web-based or automated telephone system to receive test results. While we could discuss how patients' preferences may possibly be met by such a system, we could not comment on how previous experiences might affect their preferences.

## Conclusion

The results of this study provide us with a better understanding of how patients experience notification of laboratory tests within the primary care setting. Notifying patients of test results is important for laboratory information management, and ultimately, patient safety. We believe patients can play an important role in ensuring that laboratory tests results are obtained and reviewed by providers. Asking for all laboratory tests results is a recommended strategy[24] for improving patient safety that draws patients into the feedback loop and provides a last safety net for identifying misplaced or mishandled results. Learning patients' preferences for result notification is merely one step in this important patient safety area.

## Competing interests

The author(s) declare that they have no competing interests.

## Authors' contributions

All authors contributed to the study design and conceptualization, discussed the drafts of the paper, and read and approved the final manuscript. Additionally: DB developed the study idea and conducted the data analysis. JQ conducted the interviews. CD provided technical assistance in protocol development and data analyses, and contributed to the development of the manuscript. ES contributed to the development of the manuscript. WP obtained funding for the study, helped develop the study methods, and contributed to the development of the manuscript.

## Pre-publication history

The pre-publication history for this paper can be accessed here:


